# The Role of NF1 and VCP in Frontotemporal Dementia

**DOI:** 10.1002/alz70855_107275

**Published:** 2025-12-25

**Authors:** Dariusz Pytel, Shelby Carter, William Hill, Jody F Longo

**Affiliations:** ^1^ Medical University of South Carolina, Charleston, SC, USA

## Abstract

**Background:**

Frontotemporal Dementias (FTD) presents with overlapping clinical changes in memory, language, behavior, and motor abilities. FTD is pathologically characterized by an accumulation of abnormal proteins due to a dysfunctional proteostasis network. Valosin‐containing protein (VCP) is a calcium‐dependent ATPase that functions a master regulator of protein homeostasis by modulating protein clearance, endoplasmic reticulum (ER) and mitochondria function, and protein synthesis. Neurofibromin (NF1) is a critical component of the VCP complex which promotes VCP localization to pre‐ and post‐synaptic sites where it regulates dendritic spine density by modulating local protein synthesis and autophagy. However, the functional role of this interaction has not been explored in human cases.

**Method:**

Formalin fixed paraffin embedded control and FTLD brain tissue was obtained from the Carroll A. Campbell, Jr. Neuropathology Laboratory. Samples were analyzed with ACD designed BaseScope probes for in situ hybridization and commercially available VCP, NF1, MAP2, and amyloid beta antibodies for immunohistochemical staining using standard protocols. Sections were examined under 20X, 40X and 60X magnification with an Olympus BX53 microscope.

**Result:**

Our preliminary data indicate that in control cases, neurofibromin is primarily localized in the soma, ER, and dendrites. However, in a subset of FTD cases that also exhibit Alzheimer's disease (AD) pathology, neurofibromin displays an inclusion‐like staining pattern with heavy accumulation in dystrophic neurites. NF1 and VCP co‐localization varied in entorhinal cortex neurons. Additionally, distinct staining patterns of NF1 mRNAs were observed in hippocampal neurons in disease cases. We are currently investigating how the subcellular localization of NF1 and VCP affects neuronal function and the signaling pathways involved (mTORC1/AMPK) in regulating dendritic spine density.

**Conclusion:**

In FTLD/AD patients neurofibromin and VCP are mislocalized away from synapses in neurons. Understanding how this contributes to ER‐associated proteostasis failures provides a promising avenue for therapeutic development.

